# Investigating the Influence of Antidiabetic Medications and Psychosocial Factors

**DOI:** 10.7759/cureus.60270

**Published:** 2024-05-14

**Authors:** Marc Ganz, Rena Schrier, Netanel Yomtov, Mark Spivak, Moshe Bulmash, Yisroel Appelbaum, Yehuda Gejerman, Daniel Miller

**Affiliations:** 1 Public Health Sciences, State University of New York Downstate Health Sciences University, Brooklyn, USA; 2 Internal Medicine, State University of New York Downstate Health Sciences University, Brooklyn, USA; 3 Education, New York Medical College, Brooklyn , USA; 4 Internal Medicine, Touro College of Osteopathic Medicine, New York, USA; 5 Internal Medicine, New York Medical College, Valhalla, USA; 6 Internal Medicine, Icahn School of Medicine at Mount Sinai, NYC Health + Hospitals/Queens, New York, USA

**Keywords:** mental health, depression, nhanes, sglt-2 inhibitor, diabetes

## Abstract

The relationship between type 2 diabetes mellitus (T2DM) and depression presents a significant area of medical concern, characterized by a higher incidence of depression among T2DM patients compared to the general population. This connection is not only evidenced in the prevalence of depressive symptoms in diabetic patients but also in the way these symptoms impact diabetes management. Furthermore, the influence of antidiabetic medications, especially sodium-glucose cotransporter-2 (SGLT2) inhibitors, on depression risk is a topic of ongoing research, with contrasting findings regarding the effects of drugs like metformin and pioglitazone. The aim of this study is to provide a comprehensive analysis of the relationship between T2DM and depression, focusing on the prevalence of depressive symptoms among diabetic patients, and the role of antidiabetic medications in modulating depression risk.

Methods

Utilizing data from the National Health and Nutrition Examination Survey (NHANES), we focused on individuals with T2DM. Depression status was assessed using the nine-item Patient Health Questionnaire (PHQ-9), a validated tool for evaluating depressive symptoms. Participants' depression status was categorized based on PHQ-9 composite scores. The analysis included demographic variables and the use of antidiabetic medications, with a focus on SGLT2 inhibitors. Logistic regression models adjusted for age, race/ethnicity, and BMI were employed.

Results

Our study involved 23,575 participants, of which 7,862 had T2DM. A significant difference in age and BMI was observed between diabetic and non-diabetic groups. Logistic regression analysis indicated that non-diabetic individuals had a significantly lower likelihood of depression compared to diabetic patients not on SGLT2 inhibitors. However, no statistically significant difference in depression levels was found between diabetic patients on SGLT2 inhibitors and those not on these medications.

Conclusion

These findings highlight the complex relationship between diabetes, antidiabetic medication, and depression. Notably, we found no significant impact of SGLT2 inhibitors on depression in diabetic patients, challenging previous assumptions about the role of specific antidiabetic drugs in mental health. We also revealed that older diabetic individuals reported fewer depressive symptoms, suggesting the influence of psychosocial factors and the need for age-specific depression management strategies. This research underscores the necessity of further studies to explore the nuanced effects of different antidiabetic medications on mental health outcomes, guiding toward more personalized treatment approaches for the mental health challenges in T2DM.

## Introduction

Individuals diagnosed with type 2 diabetes mellitus (T2DM) face a significantly higher risk of experiencing depression, a correlation that poses substantial clinical challenges. Major depressive episodes, characterized by persistent sadness or loss of pleasure, are prevalent in T2DM patients and can manifest through various symptoms, potentially exacerbating health outcomes through negative behavioral patterns such as poor dietary choices and reduced physical activity. The interplay between diabetes and depression involves multiple biological mechanisms, including high blood sugar levels, insulin resistance, and increased inflammatory responses, suggesting potential avenues for intervention through antidiabetic medications [1-8].

While earlier research has suggested a protective effect of oral antidiabetic drugs against depression, recent studies have presented conflicting evidence, with some pointing to metformin's potential benefit and others highlighting the antidepressant properties of pioglitazone [9-16]. Given the importance of effective management of mental health issues in T2DM, this study aims to investigate the relationship between T2DM, antidiabetic medication use, particularly sodium-glucose cotransporter-2 (SGLT2) inhibitors, and the subsequent risk of depression development, shedding light on potential therapeutic pathways to improve patient outcomes.

## Materials and methods

Data Collection

Our study analyzed data sourced from the National Health and Nutrition Examination Survey (NHANES) [17], a longitudinal project conducted by the CDC [18]. This dataset, renowned for its comprehensive health and nutritional assessments across the United States, was used to identify participants diagnosed with T2DM. Patients with depression status were identified, harnessing the nine-item Patient Health Questionnaire (PHQ-9), a rigorously validated tool designed for the assessment of depressive symptoms through participant self-report [19].

The primary outcome of interest in our study was the depression status of participants, as determined by their responses to the PHQ-9. The PHQ-9 is a well-validated tool consisting of nine questions that assess the frequency of depressive symptoms experienced over the past two weeks. Each item is scored from 0 (not at all) to 3 (nearly every day). For this study, composite scores were generated by summing the responses to these nine items. The depression status was then categorized based on these composite scores: 0-4 indicating no depression, 5-9 mild, 10-14 moderate, 15-19 moderately severe, and 20-27 severe depression. Independent variables included in the analysis were the use of antidiabetic medications, specifically distinguishing between patients on SGLT2 inhibitors and those not on these medications. Other covariates included age, race/ethnicity, and BMI. 

The statistical analysis aimed to assess the relationship between the use of specific antidiabetic medications and the risk of developing depression, as indicated by the PHQ-9 composite scores. Logistic regression models were employed to adjust for potential confounders such as age, race/ethnicity, and BMI, providing a more nuanced understanding of the diabetes-depression link. The analysis in our study was conducted using R software, version 4.2.0 (R Foundation, Vienna, Austria).

Variables

The primary variables of interest in our analysis were the depression status of individuals with T2DM, as determined by PHQ-9 composite scores, and the influence of antidiabetic medication use, with a particular emphasis on SGLT2 inhibitors. Diabetes status was identified by the response of “yes” to the question “Have you ever been told by a doctor or health professional that you have diabetes or sugar diabetes?" Depression status was identified as a response of “yes” to five or more questions in the PHQ-9 questionnaire asked within the NHANES survey. We also incorporated several demographic and clinical variables as covariates, including age, race/ethnicity, and BMI. These variables were selected based on their potential to influence both the risk of depression and the management of T2DM, offering a comprehensive perspective on the interplay between these conditions. 

Statistical analysis

The relationship between depression status and the selected variables was examined using logistic regression models. These models were carefully adjusted for age, race/ethnicity, and BMI to ensure a nuanced analysis of the data. The adjustment process was crucial for isolating the specific effects of antidiabetic medication use, particularly SGLT2 inhibitors, on depression risk among individuals with T2DM. Through this statistical approach, the study aimed to elucidate the complex dynamics at play, providing insight into potential mechanisms and pathways linking T2DM management strategies to mental health outcomes. This comprehensive statistical evaluation sought to contribute to the growing body of evidence on the intersection of physical and mental health in the context of chronic conditions such as T2DM. The study's protocol received approval from the Review Board of the Physician's Journal of Medicine, based in Queens, New York, USA, under the approval number 1123F124. 

## Results

In our study, the prevalence of diabetes was observed in 7,862 out of a total of 23,575 individuals, highlighting a significant diabetic population. Within this diabetic cohort, only 68 individuals were on SGLT2 inhibitors, a relatively small fraction compared to 7,794 not receiving this specific treatment.

A noticeable difference was observed in age and BMI between diabetic and non-diabetic groups. Diabetic patients had a higher mean age (64.78 years) and BMI (33.21), suggesting a trend toward older age and higher BMI in the diabetic population. Non-diabetic individuals exhibited a lower mean age (54.91 years) and BMI (30.09), indicating a younger and less obese demographic (Table 1).

**Table 1:** Demographic and clinical characteristics of non-diabetic and diabetic patients, stratified by SGLT2 inhibitor use SGLT2: sodium-glucose cotransporter-2

	Non-diabetic patients	Diabetic patients	Diabetics not on SGLT2 inhibitors	Diabetics on SGLT2 inhibitors
Total	15713	7862	7794	68
Mean age (years)	54.91	64.78	64.82	59.18
Mean BMI (kg/m^2^)	30.09	33.21	33.22	32.00
Race/ethnicity				
Mexican American	1412 (9%)	807 (10%)	798 (10%)	9 (13%)
Other Hispanic	1471 (9%)	861 (11%)	853 (11%)	8 (12%)
Non-Hispanic White	6751 (43%)	2751 (35%)	2728 (35%)	23 (34%)
Non-Hispanic Black	3947 (25%)	2349 (30%)	2333 (30%)	16 (24%)
Non-Hispanic Asian	1326 (8%)	640 (8%)	631 (8%)	9 (13%)
Other races - including multi-racial	806 (5%)	454 (6%)	451 (6%)	3 (4%)
Depression				
None	9957 (63%)	4518 (57%)	4476 (57%)	42 (62%)
Mild	3337 (21%)	1775 (23%)	1757 (23%)	18 (26%)
Moderate	1541 (10%)	860 (11%)	854 (12%)	6 (9%)
Moderately severe	607 (4%)	424 (5%)	423 (5%)	1 (1%)
Severe	271 (2%)	285 (4%)	284 (4%)	1 (1%)

Our analysis revealed a diverse demographic composition. Among diabetic patients, the largest groups were non-Hispanic White (2,751) and non-Hispanic Black (2,349). Non-diabetic individuals also showed a similar pattern, with non-Hispanic White (6,751) and non-Hispanic Black (3,947) forming the majority.

The logistic regression analysis, controlling for age, race/ethnicity, and BMI, revealed notable findings. The likelihood of depression in non-diabetic individuals was significantly lower compared to diabetic patients not on SGLT2 inhibitors, as indicated by a negative coefficient (-0.401970) with a p-value < 2x10^-16^. This suggests a potential impact of diabetes itself on the likelihood of depression. Age showed a negative association with depression (coefficient: -0.009407, p-value: 9.14x10^-16^), indicating that older individuals in our study were less likely to report depression. Diabetic patients on SGLT2 inhibitors did not show a statistically significant difference in depression levels compared to diabetic patients not on these medications (p-value: 0.0732) (Table 2). This suggests that the use of SGLT2 inhibitors may not have a distinct impact on depression in diabetic patients.

**Table 2:** Regression analysis of depression* scores in relation to diabetes and SGLT2 inhibitor use SGLT2: sodium-glucose cotransporter-2; CI: confidence interval * Defined as a nine-item Patient Health Questionnaire (PHQ-9) score > 10; ***: p < 0.001 (highly significant)

Variable	Coefficient (estimate)	p-value category	95% CI lower limit	95% CI upper limit
Intercept	-1.438722	< 0.001 ***	-1.6845	-1.1929
Diabetic patients on SGLT2 inhibitors	-0.769675	0.0732	-1.6117	0.0724
Diabetes patients not on SGLT2 inhibitors	-0.40197	< 0.001 ***	-0.4827	-0.3212
Age	-0.009407	< 0.001 ***	-0.0117	-0.0071
Race/ethnicity	-0.076582	< 0.001 ***	-0.1023	-0.0508
BMI	0.021552	< 0.001 ***:	0.0172	0.026

## Discussion

Individuals with T2DM show a notably higher incidence of depression compared to the general populace, with studies suggesting almost twice the probability [1-3]. Major depressive episodes, as classified by the Diagnostic And Statistical Manual Of Mental Disorders, Fifth Edition, often involve persistent sadness or loss of pleasure and may include symptoms like fatigue, cognitive shifts, appetite variation, sleep disturbances, or thoughts of self-harm [4,5]. In T2DM patients, these symptoms can manifest individually or collectively. Depression is linked to negative health behaviors in T2DM, such as suboptimal blood sugar management, poor dietary choices [6,7], and reduced physical activity adherence [8]. Despite their significance, effectively identifying and managing mental health issues in T2DM is a considerable clinical hurdle.

There's a recognized overlap between diabetes and depression, with diabetic patients facing an elevated depression risk [9]. There are three biological mechanisms that are suggested to explain this association. The first is high blood sugar levels, as studies have found a correlation between hemoglobin A1C (HBA1C) levels and depressive symptoms [10]. The second is insulin resistance. This is proposed as a connecting factor between diabetes and depression, supported by various studies at cellular, animal, and clinical levels [11]. The third is an increased inflammatory response observed in both diabetic and depressive conditions, where anti-inflammatory treatments have shown efficacy in alleviating depressive symptoms demonstrating that this may be the underlying pathophysiology [12]. Given that many antidiabetic drugs impact these pathways, it's theorized that they might also reduce depression risk in diabetic individuals [13].

Earlier research has indicated that oral antidiabetic medications can lower depression risk [14]. Yet, contrasting findings from a cross-sectional study revealed a heightened depression risk in diabetics using oral antidiabetic drugs versus those not using them [15]. Recent investigations highlighted that metformin, either alone or in combination with other drugs, was linked to a reduced occurrence of depression [16]. However, a recent meta-analysis did not support metformin's effect on depression risk, instead pointing to the antidepressant potential of pioglitazone [20]. The focus of this study is to examine the relationship between T2DM, the use of antidiabetic drugs, specifically SGLT2 inhibitors, and the subsequent risk of developing depression.

Our results highlight the complex relationship between diabetes, age, and depression. Contrary to some expectations, older age was associated with a lower likelihood of depression. This finding may reflect various psychosocial factors that confer resilience in older age, such as life experience and emotional regulation.

The absence of a significant association between SGLT2 inhibitor use and depression in diabetic patients provides an intriguing insight into the multifaceted nature of diabetes management and its psychological impacts. While SGLT2 inhibitors are a critical component of diabetes treatment for some, their role in mental health outcomes, specifically depression, requires further exploration.

The limitations of this study include several factors that may affect the generalizability and interpretation of the findings. Firstly, the use of cross-sectional data from NHANES limits our ability to establish causal relationships between T2DM, depression, and antidiabetic medications. Longitudinal studies would provide a more robust understanding of the temporal dynamics of these associations. Additionally, the reliance on self-reported data for both diabetes status and depressive symptoms introduces the possibility of recall bias and misclassification. Moreover, while we adjusted for key demographic variables in our analysis, other potential confounders such as socioeconomic status, comorbidities, and duration of diabetes were not accounted for, which may have influenced the observed relationships. Furthermore, the study population primarily consisted of individuals from the United States, potentially limiting the generalizability of our findings to other populations with different healthcare systems and cultural contexts. Lastly, the specific focus on SGLT2 inhibitors may not fully capture the broader landscape of antidiabetic medications and their potential effects on depression risk. Future research should address these limitations to provide a more comprehensive understanding of the complex interplay between T2DM, depression, and pharmacotherapy.

## Conclusions

This study has elucidated several key insights into the intricate interplay between T2DM, the use of antidiabetic medications, particularly SGLT2 inhibitors, and the risk of depression. Our findings highlight the heightened prevalence of depressive symptoms among individuals with T2DM, aligning with existing literature that indicates a bidirectional relationship between diabetes and depression. Notably, the absence of a statistically significant difference in depression levels between diabetic patients on SGLT2 inhibitors and those not on these medications challenges some preconceived notions about the role of specific antidiabetic drugs in mental health management.
